# RETRACTED: Rajkhowa et al. Activating SIRT-1 Signalling with the Mitochondrial-CoQ10 Activator Solanesol Improves Neurobehavioral and Neurochemical Defects in Ouabain-Induced Experimental Model of Bipolar Disorder. *Pharmaceuticals* 2022, *15*, 959

**DOI:** 10.3390/ph18111763

**Published:** 2025-11-20

**Authors:** Bidisha Rajkhowa, Sidharth Mehan, Pranshul Sethi, Aradhana Prajapati, Manisha Suri, Sumit Kumar, Sonalika Bhalla, Acharan S. Narula, Abdulrahman Alshammari, Metab Alharbi, Nora Alkahtani, Saeed Alghamdi, Reni Kalfin

**Affiliations:** 1 Division of Neuroscience, Department of Pharmacology, ISF College of Pharmacy, Moga 142001, India; bidishadhunu@gmail.com (B.R.); pranshulsethiptl@gmail.com (P.S.); aradhanapjt@gmail.com (A.P.); manishasuri9015@gmail.com (M.S.); sumitsrivastav223@gmail.com (S.K.); sonalikabhalla97@gmail.com (S.B.); 2 Narula Research, LLC, 107 Boulder Bluff, Chapel Hill, NC 27516, USA; acharannarula@icloud.com; 3 Department of Pharmacology and Toxicology, College of Pharmacy, King Saud University, P.O. Box 2455, Riyadh 11451, Saudi Arabia; abdalshammari@ksu.edu.sa (A.A.); mesalharbi@ksu.edu.sa (M.A.); 441203710@student.ksu.edu.sa (N.A.); smalghamdi@sfh.med.sa (S.A.); 4 Institute of Neurobiology, Bulgarian Academy of Sciences, Acad. G. Bonchev St., Block 23, 1113 Sofia, Bulgaria; reni_kalfin@abv.bg; 5 Department of Healthcare, South-West University “Neofit Rilski”, Ivan Mihailov St. 66, 2700 Blagoevgrad, Bulgaria

The journal retracts the article “Activating SIRT-1 Signalling with the Mitochondrial-CoQ10 Activator Solanesol Improves Neurobehavioral and Neurochemical Defects in Ouabain-Induced Experimental Model of Bipolar Disorder” [1] cited above.

Following publication, concerns were brought to the attention of the Editorial Office regarding the integrity of images presented in this publication [1].

Adhering to our standard procedure, the Editorial Office and Editorial Board carried out an investigation that identified partial duplication between sub-images of Figure 12 published in [1], presenting different experimental conditions. While the authors collaborated within this process, the Editorial Board has lost confidence in the reliability of the findings and has decided to retract this publication, as per MDPI’s retraction policy (https://www.mdpi.com/ethics#_bookmark30).

This retraction was approved by the Editor-in-Chief of the journal *Pharmaceuticals*.

The authors disagree with this decision.

## References

[1] Rajkhowa B., Mehan S., Sethi P., Prajapati A., Suri M., Kumar S., Bhalla S., Narula A.S., Alshammari A., Alharbi M. (2022). RETRACTED: Activating SIRT-1 Signalling with the Mitochondrial-CoQ10 Activator Solanesol Improves Neurobehavioral and Neurochemical Defects in Ouabain-Induced Experimental Model of Bipolar Disorder. Pharmaceuticals.

